# Direct Interspecies Electron Transfer Mediated by Graphene Oxide-Based Materials

**DOI:** 10.3389/fmicb.2019.03068

**Published:** 2020-01-17

**Authors:** Kensuke Igarashi, Eijiro Miyako, Souichiro Kato

**Affiliations:** ^1^Bioproduction Research Institute, National Institute of Advanced Industrial Science and Technology, Sapporo, Japan; ^2^Nanomaterials Research Institute, AIST, Tsukuba, Japan; ^3^Division of Applied Bioscience, Graduate School of Agriculture, Hokkaido University, Sapporo, Japan

**Keywords:** GO-based materials, DIET, microbial communities, *Geobacter metallireducens*, *Methanosarcina barkeri*

## Abstract

Conductive materials are known to promote direct interspecies electron transfer (DIET) by electrically bridging microbial cells. Previous studies have suggested that supplementation of graphene oxide (GO) based materials, including GO, and reduced GO (rGO), to anaerobic microbial communities, can promote DIET. This promotion mechanism is thought to be involved in electron transfer via rGO or biologically formed rGO. However, concrete evidence that rGO directly promotes DIET is still lacking. Furthermore, the effects of the physicochemical properties of GO-based materials on DIET efficiency have not been elucidated. In the current work, we investigated whether chemically and biologically reduced GO compounds can promote DIET in a defined model coculture system, and also examined the effects of surface properties on DIET-promoting efficiency. Supplementation of GO to a defined DIET coculture composed of an ethanol-oxidizing electron producer *Geobacter metallireducens* and a methane-producing electron consumer *Methanosarcina barkeri* promoted methane production from ethanol. X-ray photoelectron spectroscopy revealed that GO was reduced to rGO during cultivation by *G. metallireducens* activity. The stoichiometry of methane production from ethanol and the isotope labeling experiments clearly showed that biologically reduced GO induced DIET-mediated syntrophic methanogenesis. We also assessed the DIET-promoting efficiency of chemically reduced GO and its derivatives, including hydrophilic amine-functionalized rGO (rGO-NH_2_) and hydrophobic octadecylamine-functionalized rGO (rGO-ODA). While all tested rGO derivatives induced DIET, the rGO derivatives with higher hydrophilicity showed higher DIET-promoting efficiency. Optical microscope observation revealed that microbial cells, in particular, *G. metallireducens*, more quickly adhered to more hydrophilic GO-based materials. The superior ability to recruit microbial cells is a critical feature of the higher DIET-promoting efficiency of the hydrophilic materials. This study demonstrates that biologically and chemically reduced GO can promote DIET-mediated syntrophic methanogenesis. Our results also suggested that the surface hydrophilicity (i.e., affinity toward microbial cells) is one of the important determinants of the DIET-promoting efficiencies. These observations will provide useful guidance for the selection of conductive particles for the improvement of methanogenesis in anaerobic digesters.

## Introduction

Direct interspecies electron transfer (DIET) plays a crucial role in the anaerobic biodegradation process, in which electrons released from electron-producing microorganisms (e.g., *Geobacter* species) are transferred directly to electron-consuming microorganisms (e.g., *Methanosarcina* species) (Reguera et al., 2005; Lovley, 2011; Kato, 2015; Kouzuma et al., 2015). DIET mechanism has been extensively studied using a defined coculture system composed of electroactive microorganisms, such as *Geobacter* species and members of *Methanosarcinales* methanogens (Summers et al., 2010; Rotaru et al., 2014a, b). Rotaru et al. (2014a) used DIET coculture of *Geobacter metallireducens* and *Methanosarcina barkeri* to report basic characteristics of the model DIET coculture and determined that long-range electron transfer via pilin is necessary for DIET. They also showed that a pilin-deficient *G. metallireducens* mutant failed to establish DIET with *M. barkeri*, but an amendment of electroconductive-activated carbon permitted the mutant to establish DIET. This result suggests that conductive materials can electrically connect cells and promote DIET.

The contribution of DIET in complex microbial communities has been studied in order to evaluate the potential of DIET to increase reaction performance of anaerobic digestor and microbial fuel cells (Liu et al., 2012; Li et al., 2015; Dang et al., 2016; Lee et al., 2016; Lin et al., 2017; Tian et al., 2017). Supplementation of conductive materials to anaerobic methanogenic microbial communities promotes DIET by functioning as an electron conduit connecting electroactive cells. Various conductive materials have been assayed for DIET-promoting efficiency so far. These materials include iron oxides (Kato et al., 2012a, b; Baek et al., 2015; Yamada et al., 2015; Yin et al., 2016), activated carbon (Lee et al., 2016), biochar (Chen et al., 2014b), carbon cloth (Chen et al., 2014a), and graphite (Dang et al., 2016). Recently, electroconductive carbon nanomaterials have been extensively studied as new types of multifunctional conductive materials, owing to their unique physical and chemical characteristics (Kamran et al., 2019; Sang et al., 2019).

Graphene, a carbon nanomaterial composed of a single atomic layer of two-dimensional carbon atoms with a honeycomb structure, has attracted considerable attention because of various physicochemical properties, such as significantly high surface area, superior thermal/electrical conductivity, and photocatalytic activity (Novoselov et al., 2004; Kostarelos and Novoselov, 2014). Graphene oxide (GO) is the precursor for large-scale synthesis of graphene (Hummers and Offeman, 1958; Li et al., 2014). GO surface bears various oxygen-containing functional groups, such as hydroxyl, epoxide, diol, and carbonyl groups (Kostarelos and Novoselov, 2014). Due to these functional groups, the GO surface is hydrophilic and thus exhibits high water dispersibility and good biocompatibility, though it lacks electroconductivity (Li et al., 2014; Zhou and Liang, 2014). Chemical reduction of GO produces reduced GO (rGO), which exhibits similar physicochemical properties to pristine graphene. Thus, a number of studies have employed GO or rGO instead of pristine graphene to analyze the interactions occurring with mammalian and bacterial cells (Hu et al., 2010; Li et al., 2014; Zhou and Liang, 2014), whose potential large-scale applications could be considerable.

It has been reported that GO is biologically reduced by several microorganisms capable of extracellular electron transfer such as *Geobacter* species (Goto and Yoshida, 2016; Yoshida et al., 2016a, b) and *Shewanella oneidensis* (Jiao et al., 2011; Wang et al., 2011; Yong et al., 2014), which results in the formation of electroconductive complexes composed of rGO and microorganisms (Yong et al., 2014; Yoshida et al., 2016a). Besides, several studies have reported that supplementation of rGO to anaerobic digester promotes methane production rate and increases the population of electroactive microorganisms (e.g., *Geobacter* and *Methanosarcina* species) (Lin et al., 2017; Tian et al., 2017). These studies indicate that rGO enhances the microbial activity of anaerobic microbial communities, most likely via the promotion of DIET. However, direct evidence showing that chemically or biologically formed rGO can promote DIET has yet to be reported. Furthermore, the effects of rGO physicochemical properties on DIET efficiency remains unknown. In the present study, we examined DIET-promoting efficiency of GO using a defined DIET coculture. Besides, the effect of different physicochemical properties on DIET-promoting efficiency was assessed by chemically modified rGO, namely hydrophilic amine-functionalized rGO (rGO-NH_2_) and hydrophobic octadecylamine-functionalized rGO (rGO-ODA).

## Materials and Methods

### Microorganisms and Cultivations

*Geobacter metallireducens* (DSM7210^T^) and *M. barkeri* (DSM800^T^) were obtained from the Deutsche Sammlung von Mikroorganismen und Zellkulturen GmbH (Braunschweig, Germany). All anaerobic cultivations in this study were carried out using a sealed glass bottle (68 mL in capacity), as described previously (Kato et al., 2015), filled with 10 mL of IET-P medium under an atmosphere of 100 kPa of N_2_ + CO_2_ (80:20) at 30°C in the dark without agitation. The medium is composed of the following ingredients (per liter of distilled water): 0.3 g of KH_2_PO_4_; 1 g of NH_4_Cl; 0.1 g of MgCl_2_⋅7H_2_O; 0.08 g of CaCl_2_⋅7H_2_O; 0.6 g of NaCl; 2 g of KHCO_3_; 9.52 g of 4-(2-hydroxyethyl)-1-piperazine ethanesulfonic acid (HEPES); 1 g yeast extract; 1 g peptone; and 10 mL each of trace metal solution and vitamin solution (Sekiguchi et al., 2000). The pH of the medium was adjusted to 7.0 by adding 6 N KOH solution. *G. metallireducens* was monocultured in IET-P medium in the presence of ethanol (20 mM) as an electron donor and ferric citrate (20 mM) as an electron acceptor, as reported previously (Rotaru et al., 2014a). *M. barkeri* was monocultured in IET-P medium supplemented with sodium acetate (20 mM). Monocultures of either species were maintained by routinely transferring exponentially growing culture (ca. 5–10% volume) to the fresh medium. To establish coculture, both species from exponentially growing monocultures were inoculated into IET-P medium containing ethanol (20 mM) as the electron donor and various GO-based materials (see below). The initial cell density of *G. metallireducens* and *M. barkeri* was set at 2.5 × 10^6^ cells/mL and 2.5 × 10^5^ cells/mL, respectively, unless otherwise mentioned. Condensed inoculum prepared by centrifugation under an anaerobic atmosphere was used, if necessary. Carbon dioxide and bicarbonate ion in the medium served as potential electron acceptors (Rotaru et al., 2014a). The inoculated culture bottle was purged with N_2_ + CO_2_ (80:20) to remove carry-over methane from the inoculum and then cultivated as described above for 90 days. During cultivation, cultures were periodically sampled with 5- or 10-day intervals. All culture experiments were conducted in triplicate. Tukey’s honest significance difference test was conducted for statistical analysis.

### Preparation of GO-Based Materials Amended Media

**FIGURE 1:**
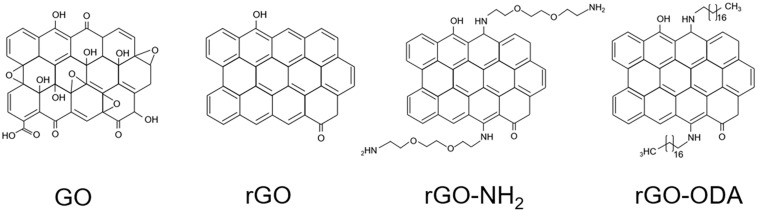
Model structures of GO-based materials used in this study.

**FIGURE 2:**
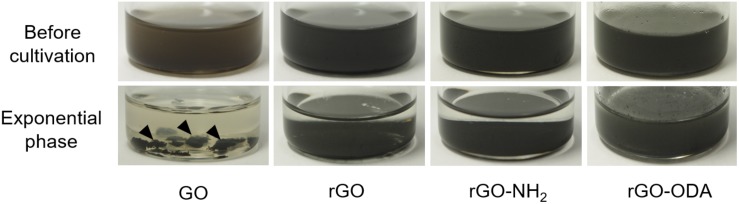
The appearance of coculture bottles containing different GO-based materials before cultivation and at the exponential phase. Arrowheads indicate self-aggregated hydrogels.

All GO-based materials were purchased from NanoInnova Technologies SL (Toledo, Spain). Figure 1 shows model structures of the GO-based materials used in this study. GO-based material was suspended in IET-P medium and vigorously mixed with a vortex mixer. The suspension was then centrifuged (6,000 × *g*, 20 min, 4°C) and resuspended in fresh IET-P medium for the following sonication process. This washing process was performed in order to remove possible inhibitory substances contained in the GO-based materials and to avoid the adsorption of trace elements that are necessary for microbial growth. Each GO-based material was suspended in IET-P medium at a concentration of 0.1% (w/v). The suspension was then sonicated using a UD-201 Ultrasonic disruptor (TOMY, Tokyo, Japan) for 1 min with 5-s intervals on ice, and dispensed to the glass bottle for cultivation. This sonication process produced medium with homogeneously suspended GO-based material (Figure 2).

### Gas Phase Analysis

For evaluating DIET efficiency, methane concentration in the gas phase was monitored periodically with a gas chromatograph GC-2014 (Shimadzu, Kyoto, Japan) equipped with a Rt-QPLOT column (30 m, 0.32 mm I.D, 10 μm F.T.; Restek, Bellefonte, PA, United States) and flame ionization detector (FID) as described previously (Kato et al., 2015).

### Liquid Phase Analysis

Liquid phase components, such as ethanol and acetate, were quantified throughout cultivation by using a high-performance liquid chromatography (HPLC) system (D-2000 LaChrom Elite HPLC system; Hitachi, Tokyo, Japan) equipped with Aminex > HPX-87H column (300 mm, 7.8 mm I.D.; Bio-Rad Laboratories, Hercules, CA, United States), a ultraviolet (UV) detector at 240 nm (L-2400, Hitachi, Tokyo, Japan), and refractive index (RI) (L-2490, Hitachi, Tokyo, Japan), as described previously (Kato et al., 2015). The liquid sample (ca. 150 μL) was periodically collected with a disposable syringe. The filtered sample (10 μL) was directly injected into the HPLC circuit and chromatographed.

### Carbon Isotope Labeling Experiment

^13^C-labeled sodium bicarbonate (NaH^13^CO_3_) was supplemented to the coculture medium with GO-amendment to obtain the final concentration of 0.5 mM. The culture bottle was cultivated as described above (section “Microorganisms and Cultivations”). Carbon isotopic compositions of methane and carbon dioxide in the gas phase of the culture bottle was determined, as previously described (Mayumi et al., 2016). In brief, gas-phase samples were taken at the initial time point of the cultivation and the stationary phase and then injected into sealed glass bottles in which the gas phase was vacuumed. Carbon isotopic compositions of methane and carbon dioxide in the gas samples were determined by gas chromatography-combustion isotope ratio mass spectrometry (GC-C-IRMS) consisting of a Trace GC Ultra, a GC IsoLink, a ConFlo IV, and a DELTA V Plus IRMS system (Thermo Fisher Scientific Inc., Waltham, MA, United States). The isotopic data were obtained as δ values in the IRMS analysis and converted to atm% by calculating (1 + δ/1000) × 1.124. The ratio of CO_2_-reduction derived methane was calculated by the method described elsewhere (Hori et al., 2011).

### Quantification of GO-Based Materials-Associated Cells

The number of cells attached to the GO-based material surface was quantified by measuring the decrease in planktonic cell density, as described below. *G. metallireducens* and/or *M. barkeri* from the exponential phase monoculture were inoculated in IET-P medium containing ethanol (20 mM) and each GO-based material under the same conditions of coculture experiment as described above (section “Microorganisms and Cultivations”), except the initial cell density of *G. metallireducens* and *M. barkeri* was set to 2.5 × 10^8^ cells/mL and 2.5 × 10^7^ cells/mL, respectively, in order to obtain significant cell densities through a direct counting method. During cultivation (0–250 h), 100 μL of culture was periodically sampled with a disposable syringe. The sample was transferred to a microcentrifuge tube and centrifuged (100 × *g*, 3 min, 4°C) to separate planktonic cells from GO-based materials-associated cells. The liquid phase containing planktonic cells was mixed with a LIVE/DEAD BacLight^TM^ Bacterial Viability Kit (LIVE/DEAD; Molecular Probes, Eugene, OR, United States) as described previously (Igarashi and Kuwabara, 2014). Cell density was determined by direct counting using a bacterial counting chamber and an optical microscope. The total number of planktonic cells was determined by counting under blue light excitation. During coculture, *G. metallireducens* and *M. barkeri* were distinguished by their morphology and based on F_420_ autofluorescence under violet light excitation, as reported previously (Igarashi and Kuwabara, 2014).

### X-Ray Photoelectron Spectroscopy (XPS)

Chemical states of GO-based materials were analyzed by XPS. Sample for XPS was collected from the culture medium by centrifugation (1,000 × *g*, 5 min, 4°C) and processed to make an XPS specimen, as described previously (Salas et al., 2010). In brief, each GO-based materials sample was rinsed with Milli-Q water three times, then washed with 80% ethanol (3–5 min), Milli-Q water (1 min), 1 N HCl (3–5 min), and rinsed with Milli-Q water at least three times. This sample was air-dried and stored in a desiccator. For the preparation of XPS specimen, the dried sample was resuspended in Milli-Q water, dispersed onto an Al plate, and dried under vacuum. XPS spectrum of C1s was obtained at the scan ranges of 275–300 eV using a PHI5000-Versa Probe (ULVAC-PHI, Inc., Kanagawa, Japan) equipped with a monochromatic Al K_α_ X-ray source. Purchased GO and rGO were analyzed and referenced as standards.

### Field-Emission Scanning Electron Microscopy (FE-SEM)

Field-Emission Scanning Electron Microscopy sample containing GO-based materials was collected from the exponentially growing culture (methane concentration ranged in 10–15 mM) and fixed with 2% (w/v) glutaraldehyde in 0.1 M sodium cacodylate buffer (pH 7.2) for 2 h at room temperature, and processed as described previously (Igarashi et al., 2016). Prepared FE-SEM specimen was mounted on an aluminum stab using carbon tape. The specimen was coated with platinum/palladium alloy with an ion sputter E102 (Hitachi, Tokyo, Japan) and observed with an FE-SEM (JSM-6330F or JSM-7800F; JEOL, Tokyo, Japan) at an acceleration voltage of 5 kV.

## Results

### Growth via GO-Mediated DIET

**FIGURE 3:**
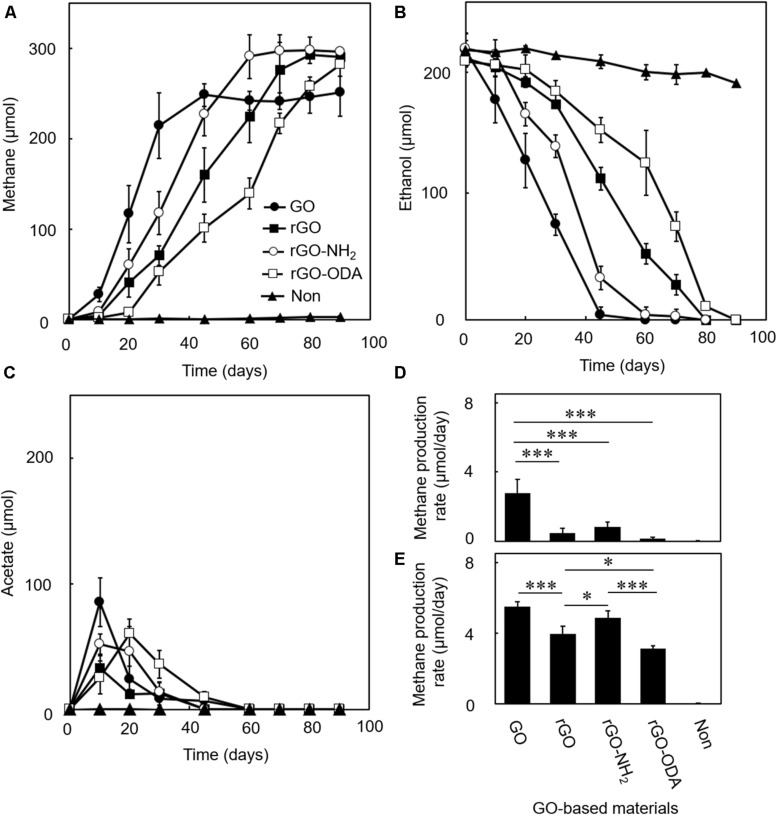
Difference in DIET-promoting efficiencies for GO-based materials. DIET cocultures of *Geobacter metallireducens* and *Methanosarcina barkeri* were conducted in the presence of each GO-based material using ethanol as the substrate. Methane **(A)**, ethanol **(B)**, and acetate **(C)** amounts were measured periodically during cultivations. Methane production rates in early- **(D)** and mid-exponential **(E)** phases were calculated from the data in **(A)**. Data are presented as the means of three independent cultures, and error bars represent standard deviations. ^∗∗∗^*p* < 0.001, ^∗^*p* < 0.05 using one-way analysis of variance with Tukey’s honest significance difference test.

We examined the DIET-promoting efficiency of GO on model coculture of *G. metallireducens* and *M. barkeri*. Amendment of GO induced methane production and ethanol consumption (Figures 3A,B), while coculture without GO (Figure 3) or monocultures of either microorganism in the presence of GO (Supplementary Figure S1) showed no methane production during the cultivation period tested (Figure 3). In the GO-amended coculture, acetate, a product of ethanol oxidation by *G. metallireducens*, was transiently accumulated in DIET-mediated ethanol degradation at the early growth phase and was nearly completely consumed during cultivation (Figure 3C). Such complete degradation of ethanol is possible only when syntrophic ethanol oxidation occurs. Theoretically, each mole of ethanol in the coculture should be converted to 1.5 mol of methane, according to the reaction schemes showing step-wise degradation of ethanol (Rotaru et al., 2014a).

Ethanol oxidation to acetate:

(1) 2CH2OH5+2HO2→2CHCOOH3+8H++8e-

CH_4_ production by acetate disproportion:

(2) 2CHCOOH3→2CH+42CO2

CH_4_ production by DIET-mediated CO_2_ reduction:

(3) CO+28H++8e→-CH+42HO2

Overall reaction (Eqs. 1 + 2 + 3):

(4) 2CH2OH5→3CH+4CO2

The GO-amended coculture produced around 248 μmol methane from 210 μmol ethanol (Figures 3A,B), which corresponded to a conversion ratio of ca. 1.2. Although the conversion ratio is slightly smaller than the expected ratio (1.5), part of the electrons generated from ethanol oxidation was expectedly consumed for GO reduction. The amount of electrons potentially consumed by GO reduction was assessed in the pure culture of *G. metallireducens* supplemented with ethanol and GO (Supplementary Figure S1A). *G. metallireducens* consumed around 65 μmol ethanol for the reduction of GO. This ethanol consumption is equal to ca. 260 μmol electron consumption (Eq. 1), and thus expected decrease in methane is ca. 33 μmol (Eq. 3), which corresponds to 89% of the observed decrease in methane (ca. 38 μmol) in GO-amended coculture. The remaining shortage was expectedly utilized for cell growth. These observations suggest that DIET-mediated syntrophic methanogenesis occurred in the GO-amended coculture.

In order to obtain further evidence for the occurrence of DIET-mediated syntrophic methanogenesis, we examined the carbon flux of the coculture by isotope labeling experiment. If ethanol is completely degraded via DIET-mediated syntrophic methanogenesis (Eq. 4), one-third of methane derives from CO_2_ reduction and the other two thirds from acetate disproportionation (acetoclastic methanogenesis). The GO-amended coculture with the supplementation of ^13^CO_2_ showed that 31 ± 9.7% of methane was produced by CO_2_ reduction, which was comparable to the expected value. This result suggests that methane production in the GO-amended coculture occurred as the result of DIET-dependent CO_2_ reduction and DIET-independent acetate disproportionation.

Graphene oxide-amended coculture exhibited a change in color, where the suspension changed from brown to black and formed self-aggregated hydrogels from the early exponential phase to the end of cultivation (Figure 2). The hydrogels were also observed in the GO-amended monoculture of *G. metallireducens*. Such hydrogels have been reported in monocultures of *Geobacter* species (Yoshida et al., 2016b) as well as in the reaction product resulting from an abiotic reduction of GO (Xu et al., 2010). Thus, it is highly probable that *G. metallireducens* in this study reduced oxygen-containing functional groups on the GO surface and induced the formation of the hydrogel.

**FIGURE 4:**
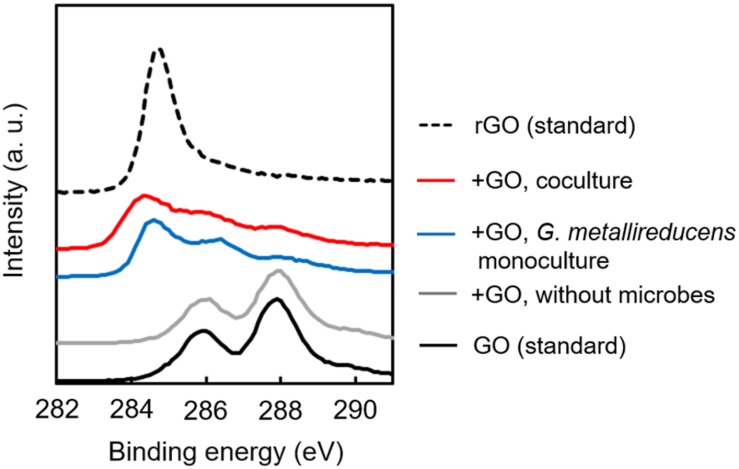
X-ray Photoelectron Spectroscopy spectra of GO-based materials. Dried specimens of GO-based materials collected from culture media before and after cultivation were analyzed by XPS. Binding energy of 284–285, C–C bond; 286, C–OH bond; 288, *C*=O bond.

In order to determine the biological reduction of GO during cultivation, XPS analysis was carried out for GO collected from exponentially growing cultures. Figure 4 summarizes the XPS spectra of GO specimens before and after cultivation. Spectrum from GO confirmed the presence of peaks for the C–OH (286 eV) and *C*=O (288 eV) bonds (Wang et al., 2011), suggesting that GO surface is highly abundant in oxygen-containing functional groups (Szabó et al., 2005). After cocultivation, these peaks were dramatically decreased, and the peak derived from the C–C bond (284–285) (Yang et al., 2009) appeared on the specimen (Figure 4). This spectrum change was also observed on GO monocultured with *G. metallireducens.* No spectrum change was observed for GO incubated without microorganism. These results conclusively suggest that *G. metallireducens* mainly contributes to the reduction of GO to rGO in coculture and also induces self-aggregation of biologically reduced GO shown in Figure 2. Collectively, these results demonstrate that biologically reduced GO can promote DIET, acting as a conduit connecting electroactive cells.

### Effects of Surface Modification of rGO

Next, we attempted to elucidate the effects of rGO surface functional groups on DIET-promoting efficiency. For the comparison, chemically reduced GO, a hydrophilic amine-functionalized rGO (rGO-NH_2_), and hydrophobic octadecylamine-functionalized rGO (rGO-ODA) were employed as model DIET-promoting GO-based materials. Cocultures with rGO and rGO-NH_2_ exhibited a stiff layer of sediment at the bottom of the culture bottle. However, coculture bottles amended with rGO-ODA did not show these macroscopic changes (Figure 2, bottom panels).

Reduced GO and its derivatives induced methane production with a near-stoichiometric ratio of 1.5 (Figure 3A), whereas monocultures with rGO did not induce methane production (Supplementary Figure S1). The DIET-promoting efficiencies of the three rGO derivatives were evaluated by methane production rates in the early- (Figure 3D) and mid-exponential phases (Figure 3E). rGO-NH_2_ showed the highest methane production rate among GO-based materials tested, which is followed by rGO and rGO-ODA. This result suggests that surface hydrophilicity is positively correlated with DIET-promoting efficiency.

### Fine Structures of GO-Based Material Surface

**FIGURE 5:**
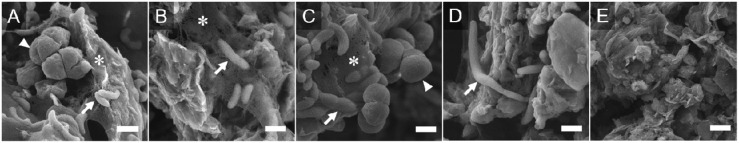
Field-Emission Scanning Electron Microscopy images of GO-based materials surfaces. Representative images of GO-based material surface morphology of exponentially growing cocultures are shown. Arrows indicate *G. metallireducens*. Arrowheads indicate *M. barkeri*. GO **(A)**; rGO **(B)**; rGO-NH_2_ **(C)**; rGO-ODA **(D)**; GO before cultivation **(E)**. Bars, 1 μm. ^∗^Exopolysaccharide-like structure.

In order to examine the fine structures of GO-based materials, surface morphologies of GO-based materials derived from exponentially growing cultures were observed under FE-SEM (Figure 5). The surface of GO and rGO-NH_2_ was enriched for both *G. metallireducens* and *M. barkeri*. In the specimens isolated from GO, rGO, and rGO-NH_2_ cocultures, cells were sometimes found to be embedded in exopolysaccharide (EPS)-like structures (Figures 5A–C; Dohnalkova et al., 2011), indicating that these microorganisms actively grew and colonized the GO-based material surface. EPS production is thought to contribute to the self-aggregation of rGO hydrogels and also to cause stiff sediment, as was observed in the rGO and rGO-NH_2_ coculture (Figure 2). Although GO has been reduced to rGO during cultivation (Figure 4) no significant change in surface morphology of cultivated GO was observed after cultivation as compared to before cultivation (Figures 5A,E).

### Measurement of Cell Attachment to GO-Based Materials

**FIGURE 6:**
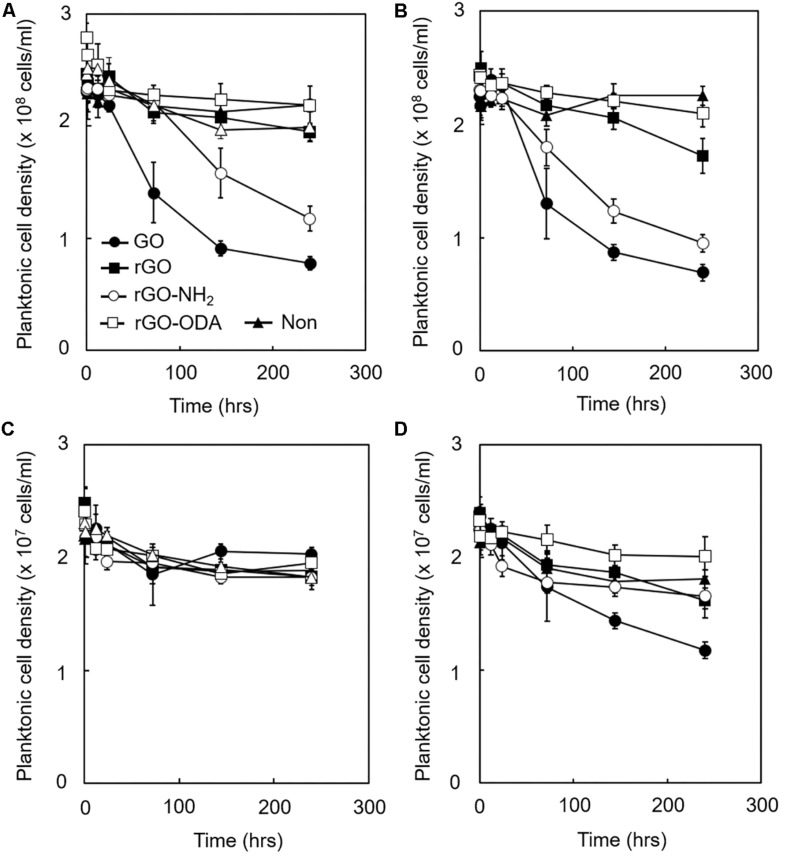
Adhesion efficiency of microbial cells to GO-based materials. Each GO-based material was supplemented with IET-P medium containing ethanol (20 mM). The planktonic cell density of *G. metallireducens* **(A,B)** and *M. barkeri* **(C,D)** is shown. Monoculture **(A,C)**. Coculture **(B,D)**. Cultivations were conducted under the same cultivation conditions as described in section “Microorganisms and Cultivations,” except the initial cell density of *G. metallireducens* and *M. barkeri* was set to 2.5 × 10^8^ cells/mL and 2.5 × 10^7^ cells/mL, respectively. Data are presented as the means of three independent cultures, and error bars represent standard deviations.

Considering these results, we hypothesized that attachment of cells to GO-based materials during the initial stage of cultivation might strongly affect DIET-promoting efficiency. Hence, we determined attachment efficiency by mixing microbial cells and each GO-based material and measuring the decrease in planktonic cell densities. The density of planktonic cells in GO-amended monoculture of *G. metallireducens* rapidly decreased within 50 h of cultivation, followed by rGO-NH_2_-, rGO-, and rGO-ODA-amended cultures, respectively (Figure 6A). This trend was also observed for the cocultures (Figure 6B). These differences in cell attachment rate reflect differences in DIET-promoting efficiency (Figure 3). In the case of *M. barkeri*, no significant change in GO-based material attachment rate was observed in monoculture (Figure 6C), probably because *M. barkeri* cannot be metabolically active in this culture condition. However, coculture with *G. metallireducens* in the presence of GO and rGO-NH_2_ showed a noticeable decrease in planktonic *M. barkeri* cell density (Figure 6D). These results indicate that attachment of *M. barkeri* to GO-based materials was strongly controlled by its DIET-partner (*G. metallireducens*), possibly via alteration of GO-based material surface properties due to surface reduction and subsequent formation of biofilm containing EPS. Most populations (>90%) of planktonic cells for microorganisms both in monoculture and coculture were viable under LIVE/DEAD observation during the entire cultivation period (250 h, data not shown). This observation indicates that the antimicrobial activity of GO-based materials (Hu et al., 2010) was negligible in our cultures, and thus the decrease we observed in planktonic cell density was not due to cell lysis, but was a result of adhesion to GO-based materials.

## Discussion

In this study, we provide clear evidence that biologically and chemically reduced GO promotes methane production by a defined model coculture of *G. metallireducens* and *M. barkeri*. The stoichiometry of methane production from ethanol and the isotope labeling experiments suggests that DIET-mediated syntrophic methanogenesis occurred in the cocultures amended with GO-based materials. This study also showed that the surface properties of GO-based materials, in particular, their hydrophilicity, significantly affect their DIET-promoting efficiency. *G. metallireducens* cells were quickly recruited onto hydrophilic GO-based materials (especially GO) before the establishment of DIET (Figure 6). It can be assumed that GO has physicochemical properties allowing for efficient recruitment and enrichment for *Geobacter* species. This assumption is supported by a previous report demonstrating selective enrichment of *Geobacter* species on GO (Yoshida et al., 2016a, b). Adhesion of *M. barkeri* was promoted in the presence of *G. metallireducens* (Figures 6C,D), suggesting that *G. metallireducens* formed environments where *M. barkeri* can establish strong affinity to the GO-based material surface probably via EPS (Figure 5) and promote DIET-dependent methane production. Considering that the GO-amended coculture showed significantly higher methanogenic rate in the early phase of syntrophic methanogenesis than other rGO derivatives (Figure 3D), attachment of *G. metallireducens*, followed by *M. barkeri*, onto the GO-based materials would be the rate-limiting step for initiation of the DIET-dependent syntrophic methanogenesis. Our results suggest that the DIET-promoting efficiency can be modulated by the addition of oxygen-containing functional groups on the GO-based material surface, while further detailed examination, including measurements of the surface hydrophilicity and *in situ* conductivity of GO-based materials, are required to evaluate the DIET-promoting efficiency quantitatively. This insight can also be applied to a wide range of conductive materials, which have been reported to enhance DIET of various microbial communities.

This study assessed DIET-promoting efficiency using a representative model DIET coculture. Hence, further experiments using different *Geobacter* species (Kato, 2017) and/or methanogens may provide additional data supporting a comprehensive understanding of the physical interaction of solid materials and microbial cells. We suspect that culture conditions also affect redox potential and surface charge of conductive materials. Detailed electrochemical analysis of conductive materials for each culture condition will help to identify the key factor(s) that specifically control the efficiency of DIET (Liu et al., 2010). In addition, metatranscriptome analysis for model DIET cultures may provide clues to elucidate mechanisms whereby electroactive microorganisms sense and attach to solid materials with different physicochemical properties.

From a practical point of view, the selection of proper materials for the promotion of DIET is crucial to establish efficient and robust DIET systems for industrial level implementation (Sasaki et al., 2018). This study showed that GO is the superior solid material for DIET promotion than other GO-based materials (Figure 3). Furthermore, we have also performed the similar coculture experiments using magnetite, that have frequently been used as a conductive material to promote DIET (Kato et al., 2012a, b; Baek et al., 2015; Yamada et al., 2015; Yin et al., 2016), instead of the GO-based materials. Methane production rate of the GO-amended coculture in the mid-exponential phase (5.5 ± 0.3 μmol/day, Figure 3E) was significantly higher than that of the magnetite-amended coculture (3.1 ± 0.2 μmol/day), suggesting that GO is the superior material for DIET promotion than conventionally utilized conductive materials. Although GO has been bulk-produced in industrial level already, large-scale utilization of GO and its derivatives in engineered environments would still be prohibited by the low cost-effectiveness. However, GO-based materials are suitable for experimentally investigating the mechanisms for DIET promotion, because it is relatively easy to modify their chemical properties by adding various functional groups (Li et al., 2014). Results obtained in this study provide fundamental knowledge for designing and selection of suitable solid materials that efficiently promote DIET. Fabrication of functionalized solids materials from cheaper solids such as iron oxides and activated carbon, which have similar physicochemical properties of GO, would increase cost-effectiveness. Furthermore, fundamental analysis using model DIET cocultures combined with an examination of pilot-scale DIET systems (Kumar et al., 2014; Zhou et al., 2019) will further promote industrial applications of DIET.

## Data Availability Statement

All datasets generated for this study are included in the article/Supplementary Material.

## Author Contributions

KI and SK designed the experiments. KI performed the experiments and wrote the initial draft of the manuscript. All authors interpreted the data and contributed to the editing of the manuscript.

## Conflict of Interest

The authors declare that the research was conducted in the absence of any commercial or financial relationships that could be construed as a potential conflict of interest.
