# Supplementary material for: Direct Interspecies Electron Transfer Mediated by Graphene Oxide-Based Materials
Source: Front Microbiol. 2020 Jan 17;10:3068. doi: 10.3389/fmicb.2019.03068 (PMC6978667; doi:10.3389/fmicb.2019.03068)

Supplementary information

Fig. SI 1. Control monocultures of *G. metallireducens* (a) and *M. barkeri* (b) were cultivated in IET-P medium containing ethanol (20 mM) and either GO or rGO. Concentrations of methane and ethanol were measured periodically during cultivations. Data are presented as the means of three independent cultures, and error bars represent standard deviations.

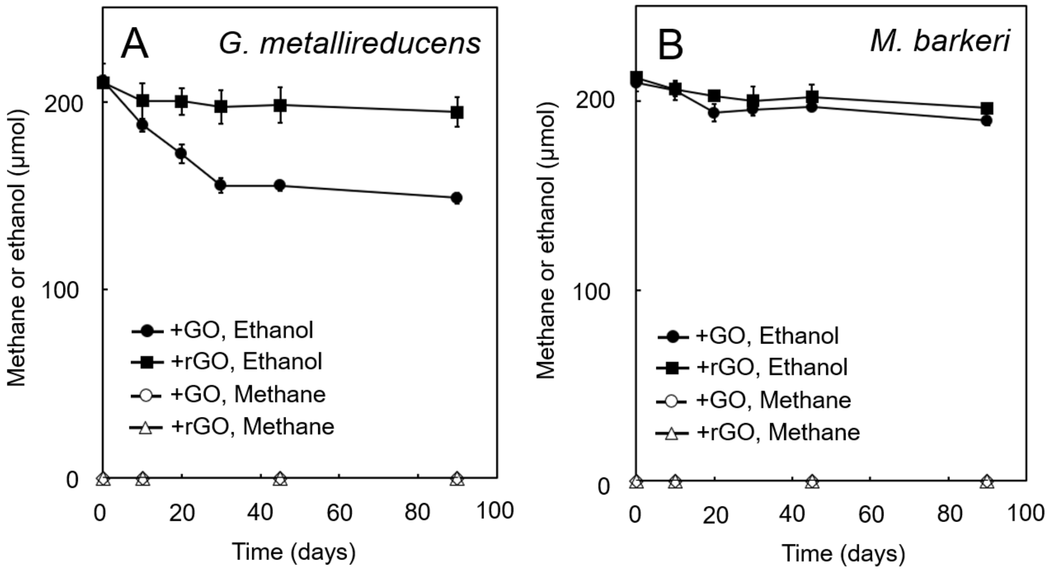

Supplement: Supplementary file 1 [file Data_Sheet_1.pdf]
